# Rapid Capillary Electrophoresis Method for Simultaneous Determination of Abemaciclib, Ribociclib, and Palbociclib in Pharmaceutical Dosage Forms: A Green Approach

**DOI:** 10.3390/molecules27217603

**Published:** 2022-11-06

**Authors:** Zvonimir Mlinarić, Lu Turković, Iva Begović, Biljana Nigović, Miranda Sertić

**Affiliations:** Department of Pharmaceutical Analysis, Faculty of Pharmacy and Biochemistry, University of Zagreb, Ante Kovačića 1, 10000 Zagreb, Croatia

**Keywords:** CDK4/6 inhibitors, abemaciclib, ribociclib, palbociclib, breast cancer, green chemistry, capillary electrophoresis

## Abstract

Advances in the treatment of HR+/HER2- breast cancer phenotype have been made with the introduction of abemaciclib, ribociclib, and palbociclib, inhibitors of cyclin D dependent kinases 4 and 6 (CDK4/6). Here, a novel, fast, cheap, and green CE method for the simultaneous determination of these three CDK4/6 inhibitors in less than 4 min is proposed for the first time. Separation was achieved by capillary zone electrophoresis in an acidic medium, in accordance with the structures of the analytes and their pKa values. The optimal pH of the running buffer was found to be 2.9. The optimal method conditions were 27.5 kV separation voltage, 30 °C, 5 s injection time under 50 mbar pressure, and 50 mM phosphate background buffer with benzimidazole as an internal standard. The developed method was validated with respect to robustness, selectivity, accuracy, precision, linearity, and limits of detection. The method was shown to be linear in the range of 10 to 100 µg mL^−1^ with correlation coefficients higher than 0.9981. A greenness assessment of the proposed method was performed, and the method was shown to be green. The validated method was successfully applied to pharmaceutical dosage forms of all CDK4/6 inhibitors.

## 1. Introduction

**Figure 1:**
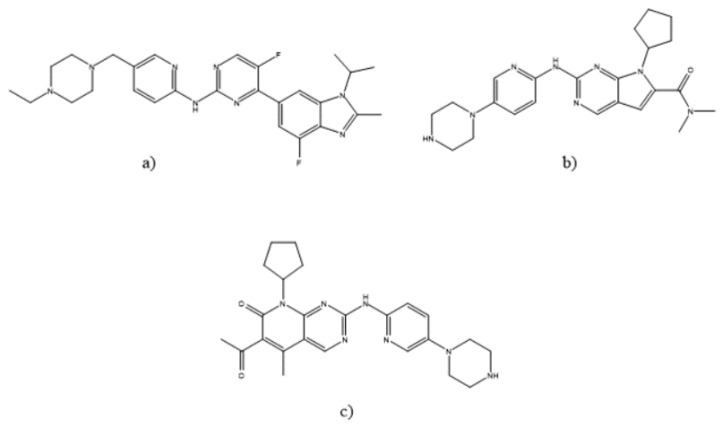
Molecular structures of (**a**) abemaciclib, (**b**) ribociclib and (**c**) palbociclib.

Breast cancer is the most common cancer in the world, with 2.3 million new cases diagnosed in 2020 [1]. This type of cancer is of major public health interest and reducing its burden on healthcare systems worldwide is of high importance. Over 70 % of breast cancers are hormone-receptor-positive (HR+) and human-epidermal-growth-factor-receptor-2-negative (HER2-) [2]. Today, the standard therapy for locally advanced or metastatic HR+/HER2- breast cancer is a combination of cyclin-D-dependent kinase 4 and 6 (CDK4/6) inhibitors with endocrine therapy because of better overall and progression-free survival [3,4,5]. Abemaciclib (ABE), ribociclib (RIB), and palbociclib (PAL) are CDK4/6 inhibitors that work by targeting overactive CDK4/6 in cancer cells and stopping their cell cycle, thereby slowing proliferation [6]. Their structures are shown in Figure 1. Analytical methods for these drugs are still scarce.

Capillary electrophoresis (CE) is a cost-efficient separation technique often considered an alternative to more widely used liquid chromatography. It offers greener, cheaper, and simpler analyses since it requires significantly smaller sample volumes and reagent consumption, and produces less waste than alternative techniques [7,8].

Furthermore, it offers great resolution and short analysis times with the potential to analyse a wide range of analytes, from small neutral and ionic molecules to large biomolecules such as proteins or nucleic acids [9].

Green chemistry is often defined as an approach to the synthesis, processing, analysis, and use of chemicals that reduce hazards to the environment and people [10]. Due to global warming, green chemistry is no longer just a new industry trend or a popular topic but rather an unavoidable perspective in every aspect of science. Furthermore, a greener approach to potentially routine analytical methods makes them cheaper, and thus more sustainable in the long term. Capillary electrophoresis has been shown to be a great tool for achieving these goals [11,12,13,14].

At the moment, there are no methods reported for simultaneous determination of ABE, RIB, and PAL, nor the individual CDK4/6 inhibitors in pharmaceutical dosage forms. There is one published method for the determination of palbociclib [15]. However, this method is an RP-HPLC method applied only to standard solutions and not to pharmaceutical formulations. Currently, only a few methods are reported for the simultaneous determination of CDK4/6 inhibitors in biological samples [16,17,18]. Based on a literature search, there are no published CE methods for the analysis of CDK4/6 inhibitors at all. Furthermore, there are no official methods or monographs for ABE, RIB, or PAL in the European Pharmacopoeia or in the United States Pharmacopoeia [19,20]. Because of the absence of monographs for these drugs in pharmacopoeias and scarce literature on this topic, the development of an analytical method for the separation and determination of CDK4/6 inhibitors in pharmaceutical dosage forms is highly required in order to control their safety, quality and efficacy. Thus, the aim of this research is to introduce a novel, fast, cheap, and green CE method for the simultaneous determination of ABE, RIB, and PAL in their pharmaceutical dosage forms.

The goal is to develop a universal method for the analysis of any CDK4/6 inhibitor without the need for the development of a separate method for each particular drug.

## 2. Results and Discussion

### 2.1. Method Development

#### 2.1.1. Effect of pH of the Running Buffer

The goal of method development was to achieve adequate separation of three CDK4/6 inhibitors and internal standard, achieving good peak shape and symmetry, while at the same time having the shortest possible analysis according to the principles of green chemistry.

Even though CDK4/6 inhibitors will never be used in combination in therapy, the goal was to develop a universal method for all of them to simplify the routine analysis of these drugs. The structures of ABE, RIB, and PAL suggest that the optimal technique for their separation would be capillary zone electrophoresis (CZE) in an acidic medium. CZE is the simplest mode of capillary electrophoresis in which the separation among analytes occurs based on their different velocities. The velocity is defined as a product of electrophoretic mobility and the applied electric field. Based on their structures, the analytes will be positively charged in a pH range from 0 to nearly 10, migrating before the electroosmotic flow (EOF), resulting in short analysis times.

**Figure 2:**
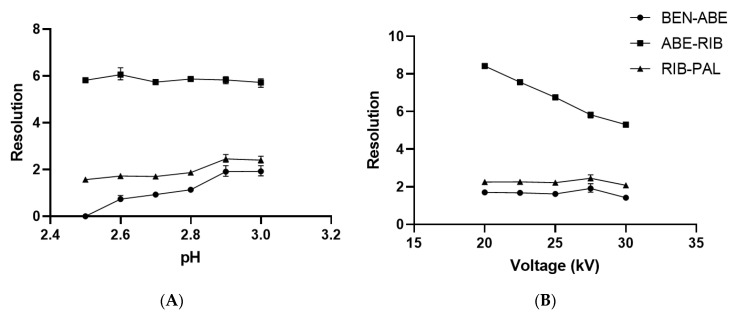
(**A**) Effect of pH of the running buffer on the resolutions of ABE, RIB, and PAL. (**B**) Effect of the applied voltage on the resolution of ABE, RIB, and PAL.

This was confirmed by evaluating the net charge of molecules in dependence on pH using Chemicalize software (Figure S1, Supplementary Material) [21]. Since ABE, RIB, and PAL are similar in size, to achieve separation between them, the pH of the running buffer for optimal separation should be that with the largest differences in net charges of the analytes at the acidic pH. This is also supported by consideration of the calculated pKa values of CDK4/6 inhibitors. Even though these molecules have many possible protonation sites in their structures, only some of them are relevant at the acidic pH. Therefore, the pKa values relevant for ABE are 1.83, 4.33, and 7.94, for RIB are 2.98, 4.31, and 8.86, and for PAL are 3.46 and 8.86. Preliminary experiments confirmed that the optimal pH should be between 2.5 and 3, since it provided good peak shapes and symmetries as well as separation. To find the optimal pH of the running buffer, a series of experiments were performed where pH was changed in the range from 2.5 to 3.0 while other separation parameters were kept constant (50 mM phosphate buffer, the temperature of 30 °C, applied voltage of 27.5 kV). With an increasing pH of the running buffer, migration times and resolutions generally increased with pH (Figure S2). Experiments with pH 2.9 and 3.0 showed similar resolutions, but pH 2.9 was chosen to be the optimal pH of the running buffer due to the fact that better peak shapes and symmetries were observed as well as slightly better resolution between ABE and RIB and RIB and PAL (Figure 2A).

#### 2.1.2. Effect of Applied Voltage and Capillary Temperature

To find the optimal voltage for the shortest separation time and the best resolution of analytes, a range of 20 to 30 kV of the applied voltage was investigated. Based on the performed experiments, increased voltage, shorter migration times and better peak shapes were observed. The analysis at 20 kV lasted 5.46 min while the analysis at 30 kV lasted only 3.15 min (Figure S3).

However, the effect on the resolution between closely migrating peaks suggested that the 27.5 kV voltage was better since the resolution was higher than 1.91 (Figure 2B), compared to the 30 kV applied voltage, which resulted in lower resolution between BEN and ABE (R = 1.42). Furthermore, at higher voltages, Joule heating in the capillary became significant and could negatively affect separation, migration times, or capillary stability. Therefore, an applied voltage of 27.5 kV was chosen as optimal because it offers the optimal separation as well as good peak shapes and symmetries with negligibly longer analysis compared to the applied voltage of 30 kV.

The effect of capillary temperature on separation and migration times was investigated at 25, 30, and 35 °C under the optimal conditions previously stated. Temperature changes the running buffer viscosity and therefore increases EOF speed, but can decrease the resolution. A capillary temperature of 30 °C was chosen as optimal since it showed the best resolution (R > 1.91) among closely migrating peaks (ABE-RIB, RIB-PAL), with negligibly longer migration times compared to 35 °C: 3.65 min instead of 3.27 min (Figures S4 and S5).

#### 2.1.3. Choosing the Internal Standard

In capillary electrophoresis, especially for quantitative analysis, it is customary to use internal standards to improve the performance of the method. One of the most critical aspects to achieving the required method performance is the injection precision, i.e., the sample volume size, since nL volumes are injected into the system using hydrodynamic or electrokinetic sample introduction techniques. There is no loop injector for such small volumes, as in HPLC for example. 

During the initial stage of method development, different molecules (diazepam, oxazepam, quinine, imidazole, benzocaine, 4-aminobenzoic acid, and benzimidazole) were tested, and benzimidazole was chosen based on its structure, pKa value, electrophoretic behaviour, and resolution from the analytes. It gave a narrow peak, migrating closely to the analytes of interest, showed similar UV absorbance, and was well resolved from the closely eluting peak of ABE.

The addition of BEN as the internal standard (IS) to the sample solution remarkably improved the precision of the injection volumes and peak areas in repeated measurements.

**Figure 3:**
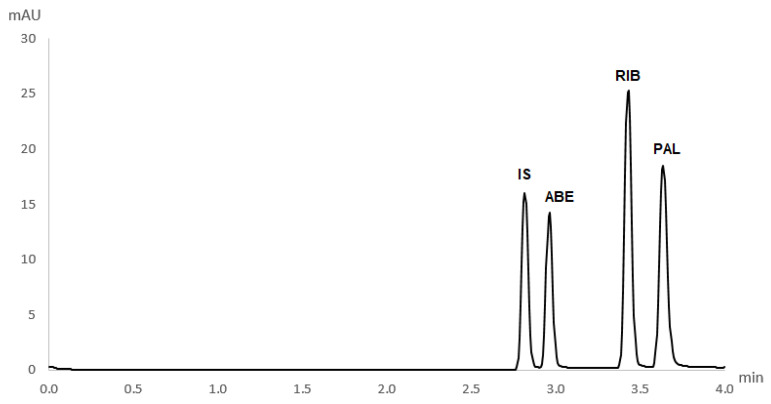
Electropherogram of standard solutions of CDK4/6 inhibitors under optimal conditions (50 mM phosphate buffer, pH 2.9, 27.5 kV, λ = 270 nm, 30 °C, injection time 5 s, pressure 50 mbar) (ABE = abemaciclib, RIB = ribociclib, PAL = palbociclib and IS = internal standard).

Each CDK4/6 inhibitor, as well as the internal standard, exhibited different absorption maxima (Figure S6). Therefore, to ensure that the sensitivity was good enough for the detection of all analytes simultaneously, the wavelength of 270 nm was chosen as optimal. Detection was performed in an extended light path capillary with a bubble cell at the detection point. This tripled the method’s sensitivity for all analytes. The electropherogram obtained in the separation of analytes under optimal conditions is presented in Figure 3. All analytes were separated in a short analysis with only 4 min of run time.

### 2.2. Method Validation

The developed CZE method for simultaneous analysis of three CDK4/6 inhibitors was validated under optimal conditions (50 mM phosphate buffer, pH 2.9, an applied voltage of 27.5 kV, a temperature of 30 °C, and injection in hydrodynamic mode of 5 s and 50 mbar), according to the ICH guidelines [22]. The selectivity of the method was determined by the assessment of peak purity by using OpenLab CDS ChemStation Edition software of standard solutions of ABE, RIB, and PAL. The purity match factor was between 999.1 and 999.8, which indicates that UV spectra from 190 to 400 nm of different parts of peaks were identical. Additionally, the method was tested on commercial drug pharmaceutical products, and peak purity factors for ABE, RIB, and PAL in their dosage forms were between 998.4 and 999.1, demonstrating that they were not co-migrating mutually nor with the excipients. Furthermore, electropherograms from standard solutions and pharmaceutical dosage forms were compared and no interferences from excipients were observed. Thus, the proposed method is selective and suitable for application to pharmaceutical dosage forms.

The linearity of the detector response was studied simultaneously by plotting ratios of corrected peak areas of each analyte and internal standard with five concentrations in the range of 10.0 to 100.0 µg mL^−1^. The calibration curves were calculated by using 1/x^2^ weighted linear regression. LOD and LOQ were calculated as concentrations where the signal-to-noise ratios of analytes were 3:1 and 10:1, respectively. The regression and correlation data as well as detection and quantitation limits are given in Table 1.

Repeatability and intermediate precision were assessed to determine the method’s precision. Intra-day precision was determined by six sequential injections of three separate standard solutions containing 50 µg mL^−1^ of ABE, RIB, PAL, and internal standard. 

Inter-day precision was performed on three consecutive days with freshly prepared BGE and standard solutions. The corrected peak areas (ratio of peak area of the analyte and of the internal standard) and the migration times of the analytes were determined, and the results are shown in Table 1. Low relative standard deviation (RSD) values indicate that the proposed method is reliable and suitable for application to real samples.

The robustness was determined by studying the influence of variation of key method parameters using a one-variable-at-a-time approach. Effects of small changes in buffer pH (2.9 ± 0.1), capillary temperature (30 ± 5 °C), and applied voltage (27.5 ± 2.5 kV) were evaluated by observing the change of relative migration times and peak areas of analytes as these parameters are varied. The results of the robustness for these parameters were expressed as RSD which was calculated as a standard deviation of the corrected migration time and corrected peak area for all peaks of interest. The changes in relative migration times and relative peak areas were small indicating that the robustness of the method was good.

The accuracy of the method was determined by comparing measured and known concentration values of the standard drug solutions. The recovery was found to be in a range from 99.9 to 104.3%, showing that the accuracy of the proposed method was good.

### 2.3. Application

**Figure 4:**
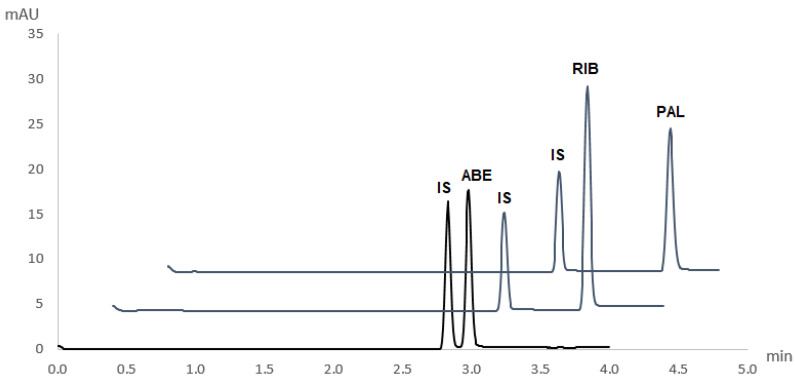
Electropherograms of ABE, RIB, and PAL pharmaceutical dosage forms under optimal conditions (50 mM phosphate buffer, pH 2.9, 27.5 kV, λ = 270 nm, 30 °C, injection time 5 s, pressure 50 mbar) (ABE = abemaciclib, RIB = ribociclib, PAL = palbociclib and IS = internal standard).

The novel and validated method for the simultaneous determination of ABE, RIB, and PAL was applied to pharmaceutical dosage forms of these drugs (Verzenios 100 mg tablets, Kisqali 200 mg tablets, and Ibrance 125 mg capsules). Obtained electropherograms are shown in Figure 4. No interferences of excipients with the analytes’ peaks were observed.

Quantitative determination was performed by comparing the peak area of a particular CDK4/6 inhibitor in its respective pharmaceutical dosage form and its corresponding peak area in a standard solution of a known concentration. Recoveries from real sample matrices were in agreement with labelled content, with mean recoveries being 99.7 ± 3.0%, 101.7 ± 2.7%, and 99.1 ± 2.6% for ABE, RIB, and PAL, respectively.

Therefore, the proposed method can be used for the quantitative determination of the pharmaceutical dosage forms of these drugs. 

Our proposed method is the first CE method for any CDK4/6 inhibitors as well as the first CE method for the simultaneous determination of ABE, RIB, and PAL. So far, there have been only a few methods reported for simultaneous determination of ABE, RIB, and PAL [16,17,18], but these are LC methods that include longer analyses and more organic solvents, making them less environmentally friendly. Although our method has lower sensitivity than LC-MS methods, this does not present any drawbacks for the determination of these drugs in pharmaceutical dosage forms. Another advantage of our method is cheaper, more applicable instrumentation compared to the expensive mass detectors. Furthermore, there are no official monographs for any of the CDK4/6 inhibitors in any pharmacopoeia; therefore, we propose our method as a fast, cheap, and green solution for the determination of these drugs in quality control for both industry and regulatory agencies.

### 2.4. Greenness of the Method

**Figure 5:**
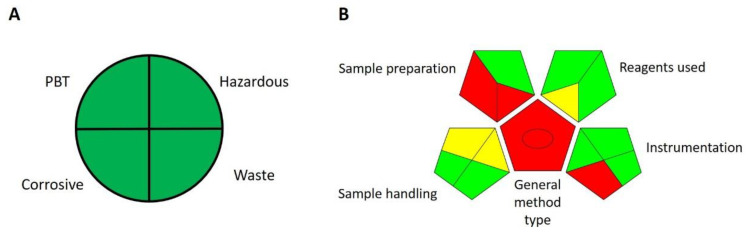
(**A**) National Environmental Methods Index’s greenness profile symbol for the proposed method. (**B**) Evaluation of greenness of our proposed method using the Green Analytical Procedure Index tool.

There are several guidelines for assessing the greenness of a particular method. A common method used for this is the National Environmental Methods Index (NEMI) developed by U.S. governmental agencies, such as the U.S. Environmental Protection Agency (EPA). NEMI considers several criteria as to whether a method uses PBT (persistent, bioaccumulative, and toxic) or hazardous chemicals as defined and listed on EPA’s toxic release inventory (TRI), whether the method uses corrosive chemicals (pH during analysis less than 2 or greater than 12) and whether the method produces more than 50 g of waste per analysis [23]. Our proposed method does not use any chemicals listed as PBT or hazardous on TRI, pH during analysis was 2.9, and waste produced per analysis was in µL. Therefore, according to NEMI’s criteria, our proposed method is perfectly green (Figure 5A). 

There is another greenness assessment method called the analytical eco-scale system which uses the Globally Harmonized System of Classification and Labelling of Chemicals (GHS) data to determine penalty points for steps, instruments, and chemicals that deviate from the ideally green analytical method [24]. The maximum score is 100 points, with a score greater than 50 representing an acceptable green analysis and a score greater than 75 representing an excellent green analysis. Our proposed method has 20 penalty points (Table 2) for using small amounts of acetonitrile, methanol, formic acid, and sodium hydroxide, while producing minimal waste. Therefore, our method has an analytical eco-scale score of 80, which means that it is an excellent green method.

Finally, there is the Green Analytical Procedure Index (GAPI), which is a tool used for the greenness assessment of analytical procedures [25]. The GAPI tool uses a pictogram composed of pentagrams to determine the greenness of each step in an analytical procedure on a colour scale from red to green. It evaluates different steps such as sample preparation and analysis, reagents and solvents, and instrumentation. Health and environmental hazards, energy expenditure, waste generation and treatment are also considered. Therefore, the GAPI tool evaluates the entire analytical procedure. Since it uses a visual representation of the greenness of each step, it can be used for fast comparison of the greenness of different analytical procedures—the greener the pictogram, the greener the method—and to spotlight weak points in a method. GAPI evaluation of our proposed method shows that it is a very green method, the only weakness being the requirement for macro-scale extraction with non-green solvents (Figure 5B).

## 3. Materials and Methods

### 3.1. Chemicals

Standards of ABE, RIB, and PAL were purchased from Toronto Research Chemicals (Toronto, ON, Canada). Benzimidazole (BEN) was purchased from Sigma-Aldrich (Burlington, MA, USA). Tablets of ABE (Verzenios) and RIB (Kisqali), as well as capsules of PAL (Ibrance), which are clinically available, were used. Running buffers were prepared from 50 mM sodium phosphate solution with pH = 2.5 and 1 M sodium hydroxide solution, both purchased from Agilent Technologies (Santa Clara, CA, USA). LC-grade acetonitrile and methanol were purchased from VWR International (Radnor, PA, USA). Formic acid was purchased from Kemika (Zagreb, Croatia). Ultra-pure water used for the preparation of all solutions and running buffers was obtained using a Milli-Q system.

### 3.2. Instrumentation

CE experiments were performed on Agilent High-Performance Capillary Electrophoresis 7100 system (Agilent Technologies, Santa Clara, CA, USA) equipped with a diode array detector. For controlling the CE system, data acquisition, and data analysis the OpenLab CDS ChemStation Edition software was used. Separations were performed on uncoated fused-silica capillaries with a 50 µm internal diameter, a total length of 48.5 cm (effective length to detector 40 cm), and an extended light path of 150 µm (Agilent Technologies, Santa Clara, CA, USA).

Before the first use, new capillaries were conditioned by rinsing with 1 M NaOH for 30 min, water for 10 min, 1 M HCl for 30 min, water for 10 min, 10 % phosphoric acid for 10 min, water for 10 min, and finally equilibrated with running buffer for 30 min. Daily conditioning was performed by rinsing with 10% phosphoric acid for 10 min, water for 10 min, and equilibration with running buffer for 20 min. Before each run, the capillary was equilibrated with a running buffer for 5 min and the capillary tip was dipped in a water vial for clean-up of both the capillary tip and the electrode before injection to achieve better reproducibility. At the end of each day, the capillary was flushed with water for 20 min and stored in water until the next usage. Running buffers were replaced every five runs.

### 3.3. CE Conditions

The optimal buffer was found to be 50 mM sodium phosphate buffer with pH 2.9, adjusted by the addition of 1 M sodium hydroxide solution. Before injection, all solutions were filtered through the 0.2 µm PTFE syringe filters (VWR International, Radnor, PA, USA). Samples were injected in the hydrodynamic mode for 5 s under the pressure of 50 mbar. Separations were carried out at the applied voltage of 27.5 kV and the temperature inside the capillary cartridge was maintained at 30 °C. Detection was performed at the wavelength of 270 nm.

### 3.4. Standard Solutions

Stock solutions of BEN, ABE and RIB were prepared by dissolving the appropriate amount of the substance in methanol to a concentration of 1 mg mL^−1^, while the stock solution of PAL was prepared by dissolving the appropriate amount of substance in a mixture of acetonitrile–water (50:50, *v/v*) with 0.1% formic acid to the final concentration of 0.25 mg mL^−1^. All stock solutions were stored in a refrigerator at 4 °C and were freshly used. In these conditions, stock solutions were found to be stable for at least 2 months.

Working standard solutions were prepared immediately before injection by dilution of the appropriate volumes of stock solutions of BEN, ABE, RIB, and PAL with acetonitrile, methanol, and water to obtain a methanol–water–acetonitrile (50:40:10, *v/v/v*) solution of BEN, ABE, RIB, and PAL of a required concentration. For optimization of the method, a solution containing 50 µg mL^−1^ of BEN, ABE, RIB, and PAL was used.

### 3.5. Tablet Solutions

Ten tablets and capsules of ABE, RIB, and PAL dosage forms were weighed and crushed in a mortar with a pestle until a fine powder was obtained. Then, in the case of ABE and RIB, an accurately weighed quantity of powder and BEN to yield a concentration of 1 mg mL^−1^ was transferred into a 50 mL calibrated flask, and 20 mL of methanol was added. In the case of PAL, an accurately weighed quantity of powder and BEN to yield a concentration of 0.25 mg mL^−1^ was transferred into a 50 mL calibrated flask, and 10 mL of water and 10 mL of acetonitrile were added. Sample solutions were then sonicated for 30 min at room temperature and filled up to the mark with methanol for ABE and RIB, and water, acetonitrile, and formic acid in the case of PAL, to reach a solution of acetonitrile–water (50:50, *v/v*) with 0.1% formic acid. Finally, solutions were filtered through 0.2 µm PTFE syringe filters (VWR, Radnor, PA, USA). Sample solutions were then analysed in the same way as standard solutions.

## 4. Conclusions

A novel, fast, cheap, and green CE method for the simultaneous determination of three CDK4/6 inhibitors in their pharmaceutical dosage forms in less than 4 min is proposed. This is the first method ever reported to be suitable for the analysis of abemaciclib, ribociclib, and palbociclib in their dosage forms. Therefore, due to the lack of official monographs in pharmacopoeias and the literature, we propose this method as an assay for the determination of these drugs in their dosage forms for industry and regulatory agencies to ensure quality, efficacy, and safety. The evaluation of the greenness of the method showed that our method is green, which ensures cheaper and safer routine use and reduces the negative impact on the environment.

## Figures and Tables

**Table 1:** Method validation parameters.

	ABE		RIB		PAL	
Concentration range (µg mL^−1^)	10.0–100		10.0–100		10.0–100	
Regression equation	*y* = 0.01832*x* + 0.01282		*y* = 0.03615*x* − 0.01534		*y* = 0.02844*x* − 0.01491	
r	0.9981		0.9984		0.9991	
LOD (µg mL^−1^)	0.25		0.08		0.11	
LOQ (µg mL^−1^)	0.83		0.26		0.36	
Intra-day precision (*n* = 6)	Migration time	RSD	Migration time	RSD	Migration time	RSD
	3.05 min	0.65	3.50 min	0.27	3.70 min	0.53
	Peak area ^a^	RSD	Peak area	RSD	Peak area	RSD
	0.92	0.94	1.74	1.05	1.52	0.33
Inter-day precision (*n* = 9)	Migration time	RSD	Migration time	RSD	Migration time	RSD
	3.01 min	1.17	3.46	0.87	3.66	0.67
	Peak area	RSD	Peak area	RSD	Peak area	RSD
	0.90	3.95	1.69	2.88	1.39	1.63

^a^ Ratio of peak areas of CDK4/6 inhibitor and internal standard.

**Table 2:** Analytical eco-scale system greenness assessment of our proposed method.

Reagents/Instruments	Number of Pictograms ^a^	Hazard ^b^	Penalty Points ^c^
Acetonitrile	2	2	4
Methanol	3	2	6
Formic acid	2	2	4
1 M NaOH	1	2	2
Waste	≤1 mL (g), no treatment		4
Technique	≤0.1 kWh per sample consumption		-
Occupational hazard	Analytical process hermetization		-
Total penalty points			20
Analytical eco-scale total score			80

^a^ Number of pictograms on the reagent packaging. ^b^ Hazard factor according to the GHS guidelines. ^c^ Penalty points are the product of the number of pictograms and hazards in case of reagents or a fixed number for a particular criteria.

## Data Availability

Data are contained within the article and supplementary material.

## Supplementary Materials

The following supporting information can be downloaded at: https://www.mdpi.com/article/10.3390/molecules27217603/s1, Figure S1: Calculated pKa values of CDK4/6 inhibitors; Figure S2: Effect of pH of the running buffer on the relative migration times of ABE, RIB, and PAL; Figure S3: Effect of applied voltage on the migration times of ABE, RIB, and PAL; Figure S4: Effect of capillary temperature on the migration times of ABE, RIB, and PAL; Figure S5: Effect of capillary temperature on the resolutions of BEN, ABE, RIB, and PAL, Figure S6: UV spectra of BEN, ABE, RIB and PAL.

## Sample Availability

Samples of the compounds ABE, RIB and PAL are available from the authors.
