# Supplementary figures and images for: Rapid Capillary Electrophoresis Method for Simultaneous Determination of Abemaciclib, Ribociclib, and Palbociclib in Pharmaceutical Dosage Forms: A Green Approach
Source: Molecules. 2022 Nov 6;27(21):7603. doi: 10.3390/molecules27217603 (PMC9657767; doi:10.3390/molecules27217603)

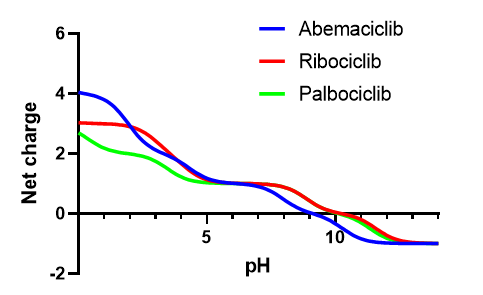

Supplement: Supplementary file 1 [file molecules-27-07603-s001.zip › Figure S1.png]

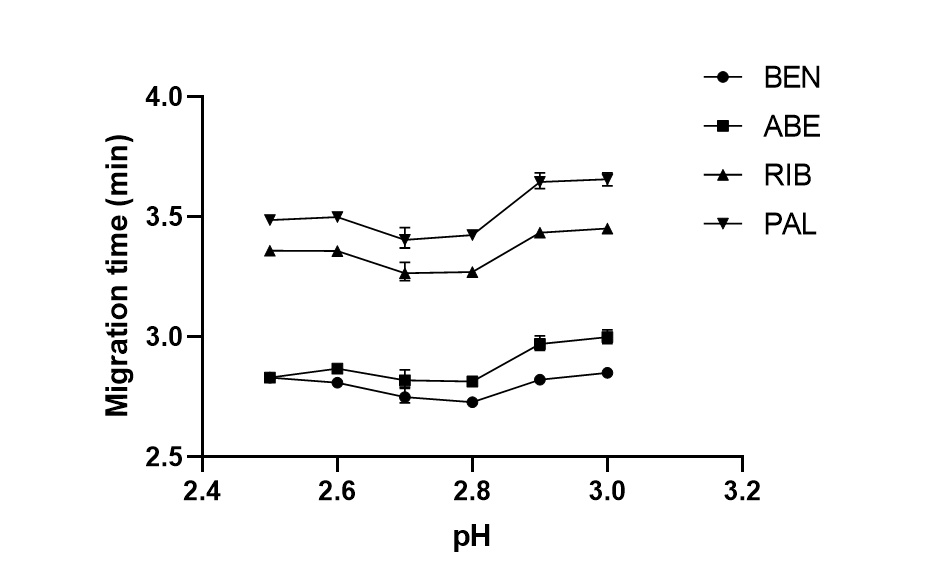

Supplement: Supplementary file 1 [file molecules-27-07603-s001.zip › Figure S2.png]

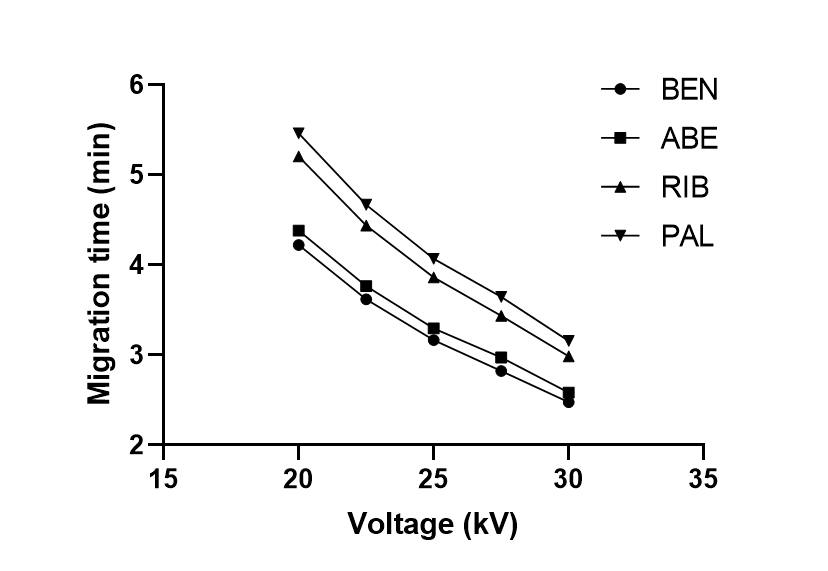

Supplement: Supplementary file 1 [file molecules-27-07603-s001.zip › Figure S3.png]

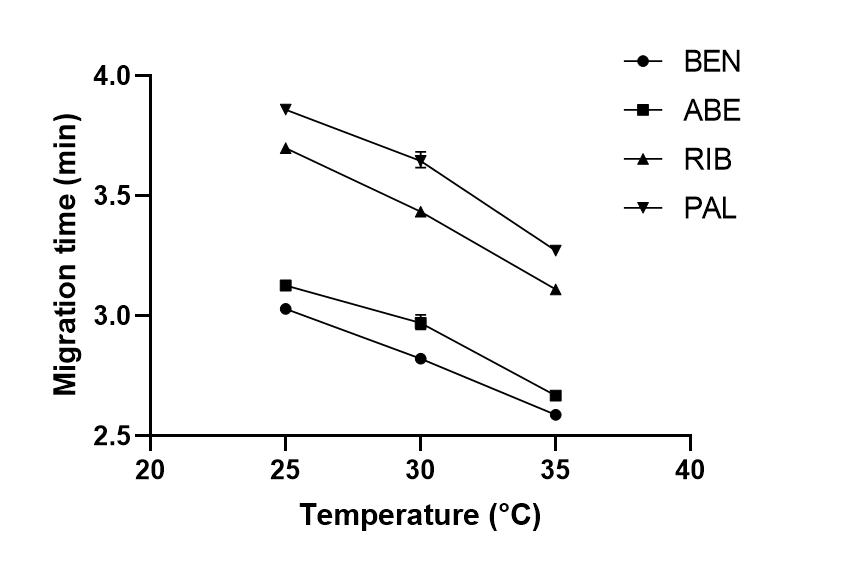

Supplement: Supplementary file 1 [file molecules-27-07603-s001.zip › Figure S4.png]

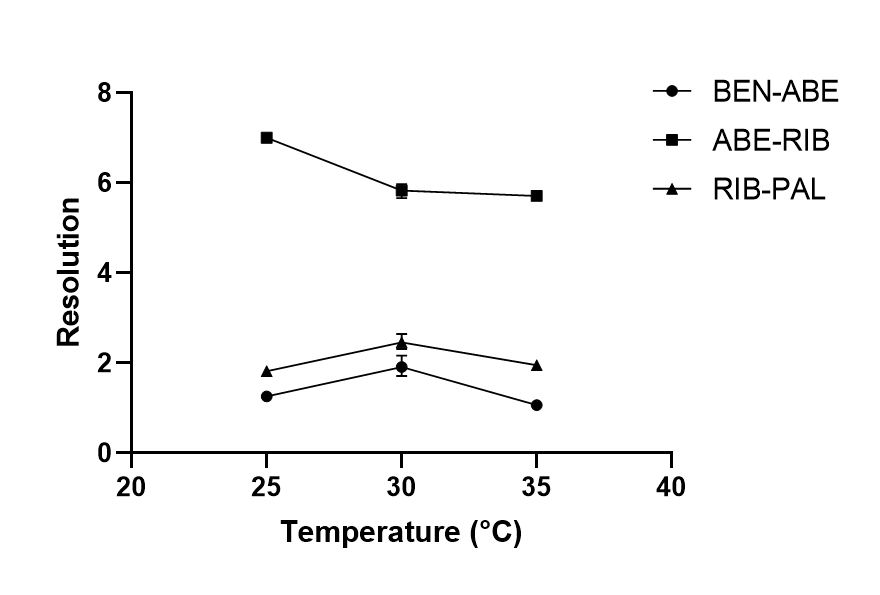

Supplement: Supplementary file 1 [file molecules-27-07603-s001.zip › Figure S5.png]

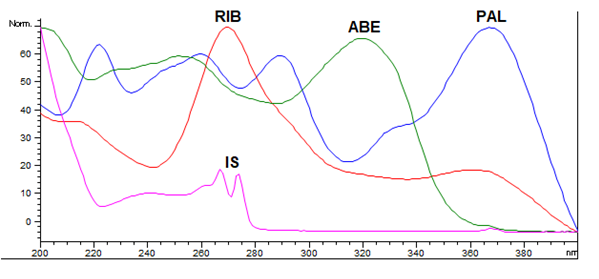

Supplement: Supplementary file 1 [file molecules-27-07603-s001.zip › Figure S6.png]
